# Clinical applications of magnetoencephalography in epilepsy

**DOI:** 10.4103/0972-2327.61271

**Published:** 2010

**Authors:** Amit Ray, Susan M. Bowyer

**Affiliations:** 1 Comprehensive Epilepsy Program, Henry Ford Hospital, Detroit MI; 2 New York University Epilepsy Center, New York NY, USA; 3 Department of Neurology, Wayne State University Detroit MI; 4 Department of Physics, Oakland University, Rochester MI, USA

**Keywords:** Magnetoencephalography, epilepsy surgery, pre-surgical evaluation, magnetic source localization, functional brain mapping

## Abstract

Magnetoencehalography (MEG) is being used with increased frequency in the pre-surgical evaluation of patients with epilepsy. One of the major advantages of this technique over the EEG is the lack of distortion of MEG signals by the skull and intervening soft tissue. In addition, the MEG preferentially records activity from tangential sources thus recording activity predominantly from sulci, which is not contaminated by activity from apical gyral (radial) sources. While the MEG is probably more sensitive than the EEG in detecting interictal spikes, especially in the some locations such as the superficial frontal cortex and the lateral temporal neocortex, both techniques are usually complementary to each other. The diagnostic accuracy of MEG source localization is usually better as compared to scalp EEG localization. Functional localization of eloquent cortex is another major application of the MEG. The combination of high spatial and temporal resolution of this technique makes it an extremely helpful tool for accurate localization of visual, somatosensory and auditory cortices as well as complex cognitive functions like language. Potential future applications include lateralization of memory function.

## Introduction

Magnetoencephalography (MEG) is a technique that helps localize sources of electrical activity within the human brain by non-invasively measuring the magnetic fields arising from such activity. [1–4] Though a relatively new technique, MEG is rapidly becoming an invaluable, often indispensable tool in the diagnostic armamentarium of the neurophysiologist. While the major applications of this test are in the field of epilepsy especially with regards to functional localization and localization of the epileptic focus, other conditions in which it might prove useful include autism,[5] stroke, [6] schizophrenia,[7] and Parkinsonism.[8] After a brief initial overview of the basic science and methodology of MEG, this review will concentrate on the major clinical applications of this technique in epilepsy.

## Basic Principles

**Figure 1:**
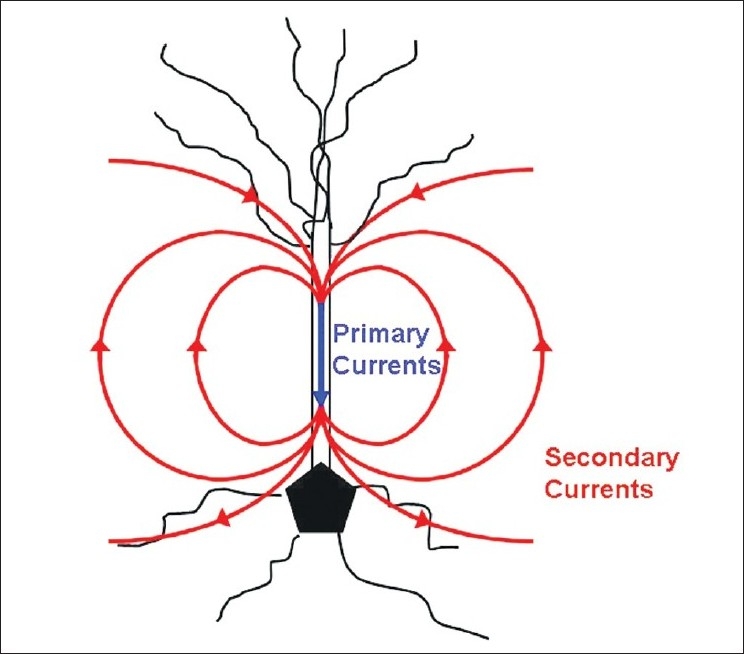
A neuronal pyramidal cell is seen in this image with primary (intracellular) currents and secondary (volume/extracellular) currents. Primary currents are depicted in blue and secondary currents in red. MEG signals are a measure of the intracellular current produced by the apical dendrites and therefore more apt to accurately represent the actual source generator. EEG signals recorded at the scalp electrodes are a measure of extracellular currents

Brain neuronal activity generates electrical currents, which in turn generate electrical field potentials detectable by the electroencephalogram (EEG). These neuronal currents also produce a magnetic field that is detectable by MEG. However, while the EEG measures extra cellular currents, the MEG is a measure of the intracellular currents generated by the apical dendrites [Figure 1].

The pyramidal cells in the brain, which are oriented perpendicular to the cortical surface, are the sources of both EEG and the MEG. As per the right hand rule of physics, the magnetic field generated by the neuronal current encircles the generating neuron at right angles to its long axis. Hence, for tangentially oriented neurons, the magnetic field exits the head at one point and re-enters it at another thus producing a minima and a maxima. This does not hold true for neurons radially oriented to the cortical surface, in which case the magnetic field produced does not exit the head and thus is undetectable by external sensors.[9] The MEG thus cannot detect sources of current which are oriented in a perfectly radial fashion, such as those generated by neurons present on the apical gyri. This apparent limitation may have only limited practical application as such perfectly radial sources are extremely rare.

**Figure 2:**
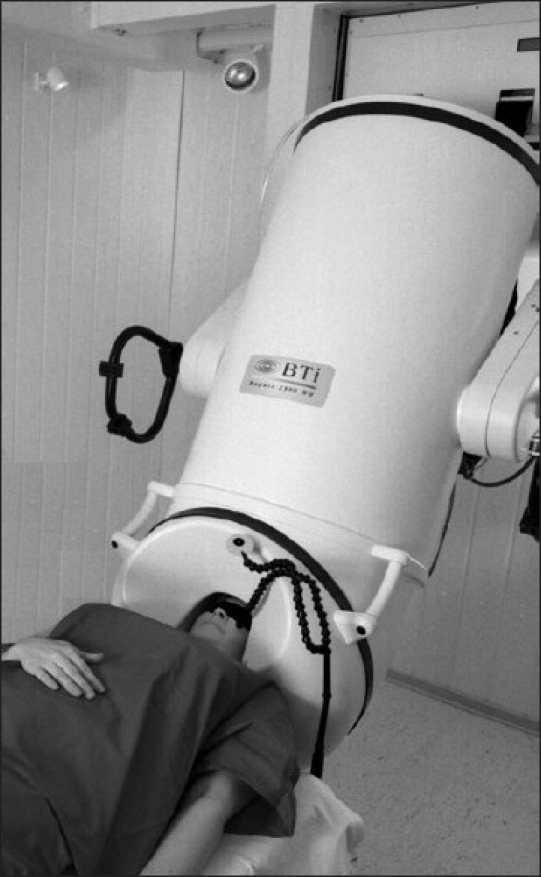
Patient in MEG machine. The cylinder contains the liquid helium. The SQUID sensors in the machine are located in close proximity to the patient's head

The magnetic signals, thus generated by the brain's neuronal activity are exceedingly small [10] on the order of a few pico to femto Tesla (10^-12^ to 10^-15^ T). In comparison to other intrinsic magnetic fields in the body as well as the atmosphere, this is miniscule (the field generated by the heart is 100 times greater than the magnetic fields generated by the brain; the magnetic field of the earth is approximately a billion times greater). The only way to measure such small magnetic fields is by the use of superconducting quantum interference devices (SQUID) bathed in liquid helium to keep them at superconducting temperatures. In the absence of these SQUIDs, the MEG signal would be lost in just attempting to overcome the impedance of the recording coil present in the MEG sensor. The combination of the recording coil and the SQUIDs at superconducting temperatures converts the tiny magnetic fields into an electric current and subsequently an output amplified voltage as in the EEG [Figure 2]. The problem of ambient noise generated by other external magnetic fields in the environment is generally overcome by using a magnetically shielded room and reference channels.

One of the major advantages of the MEG over the EEG is that the skull and the intervening soft tissues between the brain and the scalp do not distort the MEG signals. Magnetic fields pass through bone, soft tissue, and body fluid unattenuated. This is in contrast to the EEG signals which are significantly affected by the presence of skull and other soft tissues. These tissues distort the electric fields as they have different resistivities and will change the electric field as it flows through them. Another potential advantage of MEG over the EEG includes the selectivity of the MEG for tangential sources, as has been discussed earlier, thus recording activity predominantly from sulci, which is not contaminated by activity from apical gyral (radial) sources.[11] As will be discussed subsequently, there is currently no other technique that provides the combination of millisecond temporal resolution and high spatial resolution (<5 mm) in a safe, noninvasive imaging modality, other than MEG. The relatively high cost of MEG equipment and support infrastructure (including the magnetically shielded room), which is approximately of the order of 2 million US dollars is probably one of the major reasons why this technique has not become more popular. Contrast this with the typical cost for high-quality 32 channel digital video-EEG machines which cost in the range of 30,000 to 35,000 US dollars.

## Localization of the Epileptic Focus

**Figure 3:**
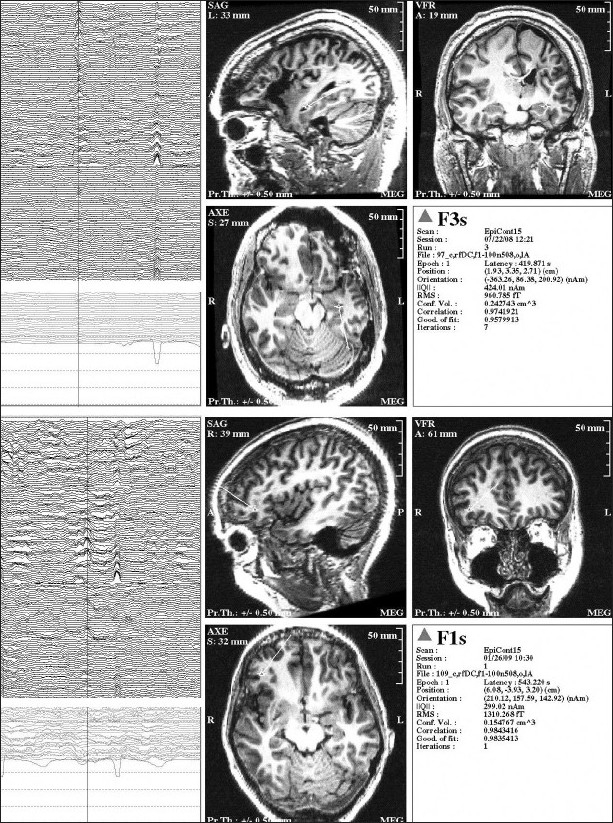
Inter-ictal spike as seen on MEG and EEG. The top 2 images are from a patient with left temporal lobe epilepsy, while the bottom 2 images are from a different patient with right frontal epilepsy. The panel on the left shows the actual MEG and EEG recordings; the channels in the top portion of each image represent the MEG, while the bottom channels are EEG tracings. The panel on the right shows the MEG equivalent current dipole source localization for the particular inter-ictal spike highlighted by the cursor

Localization of the epileptic focus using MEG, relies primarily on dipole source modeling of waveforms generated.[1,2] While this technique has typically been used to model interictal spikes, similar methods can be used to model other epileptic activity e.g. seizures. The most common dipole technique for localization of interictal spikes is the single equivalent current dipole (ECD) technique.[12] Localization is performed by comparing the measured field pattern on the MEG with a simulated field pattern which is estimated (modeled) by a computer. The latter uses several point sources placed at various positions and orientations inside a sphere located within the skull and estimates the possible fields produced by these sources (the forward solution). The inverse solution is when we predict where the current source is located, from the magnetic fields detected outside of the head. A leastsquares methodology (details are out of scope of this review) is used for finding a solution i.e. to find a point source that will accurately model the measured magnetic field. It is important to note that the real generators of the magnetic field potentials are not the point sources that have been modeled by the ECD technique but instead are large collections of synchronously firing neurons in the cerebral cortex that extend over areas of several square centimeters.[9] The point dipole [Figure 3] is just a mathematical model, which represents the sum total of activity produced by the actual source. This equivalent dipole source should likely reside at the center of the actual source and should have a similar orientation as the neurons in the real source. The equivalent source dipoles are subsequently overlaid on the patient's brain MRI. This combination of MRI and MEG source modeling is also known as magnetic source imaging (MSI). Although somewhat simplistic, the ECD model is very useful for many clinical situations but works best with stationary, non-distributed sources e.g. stable, non-propagating spikes, early components of somatosensory, auditory, and visual evoked responses [Figure 2].

Other source analysis techniques use distributed source models that assume multiple sources in the human brain are simultaneously active. This type of modeling allows extended patterns of currents to be mapped. Examples of this type of brain activity include seizures, which by definition evolve in frequency as well as location and thus usually do not remain localized to a single point. Other examples include propagating spikes, cortical activation from language, memory or other higher cognitive functioning processes. Various modeling techniques exist for this type of source analysis. A brief overview of these techniques is provided here; detailed discussion is out of the scope of this review.

An extension of the ECD technique is the multi-dipole ECD technique where more than one dipole can be fit. Multiple Signal Classification (MUSIC) and Recursively Applied and Projected-Multiple Signal Classification (RAP-MUSIC) are examples of such multi-dipole ECD [13,14] techniques. Current distribution analysis techniques (distributed source models) like the minimum norm estimates (L1, and L2) (used at Massachusetts General Hospital)[15] or MR-FOCUSS [16] which was developed and used in our lab at Henry Ford Hospital are routinely used for mapping seizures as well as cognitive functioning.[17] In this type of analysis it is assumed that the sources have a continuous distribution in the cortex. A model of the brain, with thousands of tiny dipoles seeded in the gray matter, is used to determine the most probable current distribution of these dipoles to explain the measured data. Unlike the ECD technique which can consider only one of the dipoles on at any given point in time, these techniques take into account many simultaneously active dipoles all across the cortex. The current distribution results can be displayed at each millisecond indicating the estimated strength of the activation or a related statistic as a function of time. These millisecond images can be strung together to create movies of brain activation. Low resolution electromagnetic tomography (LORETA), standardized low resolution electromagnetic tomorgraphy (sLORETA), and dynamic statistical mapping (dSPM) are some examples of these methods.[19–20]

Another type analysis used in MEG is spatial filtering. The most common is the beamformer. A beamformer is a set of spatial filters that linearly integrate information over multiple spatially distributed sensors. The basic principle of beamformer design is to allow the neuronal signal of interest to pass through in certain source locations and orientations, called pass-bands, while suppressing noise or unwanted signal in other source locations or orientations, called stop-bands.[22]

## Sensitivity of MEG in Detecting Inter-Ictal Spikes

In patients with temporal lobe epilepsy (TLE), it is known that cortical areas of at least 10 square cm (typically of the order of 20-30 cm^2^) have to be synchronously activated for spikes to be detected on the scalp EEG.[23] However, using studies involving MEG and simultaneous intracranial EEG, it has been estimated that at least 6-8 square cm of temporal lobe neocortex needs to be activated for spike detection by the[24–26] MEG. Much smaller areas of activation are required for MEG spike detection in the lateral frontal cortex (3-4 square cm). Thus, while it certainly appears that MEG is more sensitive than scalp EEG in detecting epileptic spikes, the lack of systematic simultaneous studies of MEG, intracranial EEG and scalp EEG make direct comparisons difficult.

The MEG however offers no major advantage over scalp EEG for detection of deep sources. In fact studies comparing MEG and intracranial EEG have clearly demonstrated that spikes emanating from deep structures detectable by the latter (e.g. hippocampal spikes detected by subdural and depth electrodes) are not visualized on the MEG.[25–28] Of course, in cases where the brain or skull anatomy is disrupted (e.g. breach defect in skull), the MEG does have distinct advantages and less distortion as compared to the scalp EEG.

The practical implications of this apparent increased sensitivity of the MEG over the scalp EEG are not entirely clear. Most interictal spikes are seen by both modalities i.e. the scalp EEG and the MEG.[29–31] However, there are a small number of spikes seen exclusively with one technique as compared to the other. As has been discussed earlier, the EEG detects both radial and tangential sources, while the MEG detects primarily tangential sources of field. While this lower sensitivity of the MEG to radial sources makes this an easier source to model, this is also a potential drawback. The MEG may not detect current sources that are for instance purely limited to the crowns of the apical gyri. However, it is rare to find this kind of localized source, and thus this somewhat theoretical lack of sensitivity is likely to have only limited practical implications. Conversely, sources that are in the convexities of the brain (e.g. lateral frontal neocortex) may be seen better by MEG as compared to the EEG. Sources in sulcal banks such as the sylvian fissure or the interparietal sulcus may also be better detected by MEG, as these produce predominantly tangential dipoles.

Iwasaki *et al*.[31] in their concomitant scalp EEG--MEG study detected interictal spikes on both modalities in 31 of 43 patients, MEG alone in 8 patients, and EEG alone in 1 patient. No interictal spikes were detected in three patients with either modality. The accuracy of localization of spikes was greatest when these were seen on both modalities. In the few cases when spikes were seen only on one modality, the localization accuracy was less certain especially if the total number of spikes was few. Thus, while the MEG might be somewhat more sensitive than the scalp EEG in detecting interictal spikes, the clinical utility of this increased sensitivity is not entirely clear as spikes (especially if they are infrequent) seen only in one modality might not be well localizing of the epileptic focus. In the aforementioned study by Iwasaki *et al*. two of eight patients who had only MEG spikes (in both cases localized to the parietal lobe) had a relatively poor outcome with regards to seizure freedom as opposed to patients with spikes seen on both modalities, who typically had a good post-resection outcome. This suggests that the lack of convergence of data (i.e. no common EEG-MEG spikes) may decrease the reliability of the information and result in less favorable outcome after surgery.

In conclusion, most studies suggest that MEG is more sensitive for spike detection in some areas of the brain compared with scalp EEG, such as the superficial frontal lobe and the lateral temporal neocortex. This suggests that MEG is more likely to be helpful in neocortical epilepsy. Considering that these are the types of epilepsy that may be most difficult to localize with the scalp EEG, MEG may be a very useful tool. However, most studies of simultaneous MEG and scalp EEG suggest that both techniques are complementary in epileptic spike detection

## MEG Accuracy

Using implanted dipoles (created by using special intracranial EEG electrodes) in the brain, MEG-predicted localizations were within 4 mm of the actual location of the source. MEG predicted localizations were within 1-2 mm of the actual source in mesial and basal temporal brain regions as compared to the infero-lateral temporal region where predicted localizations were within approximately 4 mm of actual localization identified by using the aforementioned implanted dipoles.[32,33]

Simultaneous intracranial EEG--MEG studies have been helpful to validate the localizing value of MEG. Though limited in number the few available studies [24–26] seem to suggest that MEG spike localization is approximately concordant with spike localization using intracranial EEG. This could be extrapolated to suggest that MEG spike localization is more accurate than scalp EEG spike localization. However, the absence of simultaneous studies of scalp EEG, intracranial EEG, and MEG make it impossible to prove this statement. Moreover, it is well recognized that multiple populations of inter-ictal spikes are visible on the intracranial EEG, not all of which are of localizing value.[34] In addition, the intracranial EEG samples only a small area of brain electrical activity, as this is limited by the extent of implantation of intracranial electrodes. Both these statements and other studies suggest that intracranial EEG interictal spikes might be poorly localizing and thus of little value as a gold-standard benchmark for comparison.

Most of the evidence regarding the localizing value of MEG inter-ictal spikes is indirect i.e. MEG spike dipoles clustered at or around a distinct lesion seen on the brain MRI and subsequent removal of this lesion resulting in seizure freedom. This is best appreciated in intrinsically epileptogenic lesions like tumors.[35,36] In some cases involving focal cortical dysplasias where the actual epileptogenic tissue might extend much further than the lesion evident on brain MRI, MEG dipoles may also extend further away from the actual lesion and are concordant with spikes evident on electrocorticography. Removal of these concordant zones of irritability on MEG and intracranial EEG, even in areas that appear normal on brain MRI, results in seizure freedom, while incomplete removal (possibly secondary to surrounding eloquent cortex) may result in incomplete seizure control. In addition, MEG may also help detect abnormal areas in cryptogenic epilepsy[37] (i.e. with normal brain MRI) and help direct appropriate placement of intracranial EEG electrodes in these cases.

Intracranial EEG seizure onsets are the currently accepted gold standard for seizure localization in most cases. Multiple studies have shown MEG localizations to be concordant with the area of seizure onset as evident on the intracranial EEG[38–40]

## Clinical Utility of MEG

Studies comparing the value of MEG, scalp video-EEG (V-EEG), and brain MRI in localization of the epileptic focus have suggested that MEG is definitely of value in the pre-surgical evaluation. The sensitivity[41] of an interictal MEG study for detecting clinically significant epileptiform activity was approximately 80%. While the MEG and V-EEG results were equivalent in 32.3% of the cases, additional localization information was obtained using MEG in 40% of the patients. MEG helped localize the resected region in 72.3% patients as compared to 40% that were localized with V-EEG. More importantly, MEG contributed to the localization of the resected region in 58.8% of the patients with a non-localizing V-EEG study and 72.8% of the patients for whom V-EEG only partially identified the resected zone. Other studies[42,43] have reported similar results.

## Ictal MEG

It is not an uncommon misconception to say “that only interictal spikes can be recorded with MEG, while seizures cannot be recorded by this technique.” It is mainly logisticaconsiderations that have prevented this technique from becoming more popular for recording of ictal events. The lack of accurately predicting the timing of a seizure and thus obtaining an MEG recording during the event as well as safety issues of a patient having a seizure in the MEG machine have been major limitations. In addition, the muscle and movement artifacts during a seizure also contribute to a poor signal-to-noise ratio. To circumvent this last problem, it is important to try and analyze the rhythms prior to obvious clinical manifestations. There is also the question that MEG may detect only propagated rhythms as opposed to the rhythms at the area of actual seizure onset. This may be especially true of hippocampal seizure onsets, which are usually undetectable on the scalp at the time of onset but only detectable when they propagate to the lateral temporal neocortex. However, this problem is not unique to MEG, and also holds true for the scalp EEG, which is also unable to detect hippocampal rhythms until they propagate outside the hippocampus and recruit a sufficient amount of temporal neocortex. Our personal experience of 13 patients who had a seizure during the MEG recording showed the ictal MEG to be well localizing of the ictal onset zone in all 13 patients, using the MR-FOCUSS technique of localization.[44]

In another study[45] of 20 patients with neocortical epilepsy, successful ictal MEG recordings were made in 6 patients. In one patient, a seizure was captured but movement artifact made MEG recordings impossible. As determined by invasive EEG recording and postsurgical outcome, ictal MEG provided localizing information that was superior to interictal MEG in three of the six patients. Localization of ictal onset by MEG was at least equivalent to invasive EEG in five of the six patients, and was superior in two patients as determined by postsurgical outcome. Other series have also found consistent localization of ictal MEG with IC-EEG and good surgical outcomes.[46,47]

## High Frequency Oscillations and MEG

Recent reports,[48–50] using invasive intracranial recordings, have suggested that high-frequency oscillations (HFO's) can be used to accurately localize the epileptogenic zone. Fast ripples (HFO's with a frequency of greater than 200 Hz) have been particularly helpful in localization. The MEG can also be helpful[51] in recording similar HFO's. In a recent[52] study of 30 children with epilepsy, 26 patients had HFO activity recorded by MEG and MEG source localizations of HFO activity were found to be concordant with intracranial EEG in 9 of 11 (82%) patients who had epilepsy surgery. In addition, the HFO activity was concordant with MRI lesion in 21 of 30 (70%) patients.

## Functional Brain Mapping Using MEG

Localization of functional areas of the brain, also called brain mapping, is an important application of MEG. The physiologic basis of MEG brain mapping is similar to that of stimulus evoked electrical potentials detected at the scalp. Stimuli can include somatosensory, auditory, visual, etc. The neuronal currents generated by these stimuli at the brain, apart from producing electrical potentials, also generate magnetic detectable by the MEG. Synchronization of task timing with responses allows for mapping of eloquent functional cortex. Just as with electrical-evoked responses, averaging over multiple stimulus epochs results in better signal-to-noise ratios and consequently better MEG recordings.

The combination of extremely high spatial (mm) and temporal resolution (ms) of the MEG technique as well as the relative lack of attenuation of signals by intervening skull and soft tissues makes this technique extremely conducive for this purpose. As a result of these advantages MEG can be used for cortical localization of relatively simple functions as well as complex cognitive functions like language, within an accuracy of a few mm, which require sequential temporal activation of multiple cortical areas.

**Figure 4:**
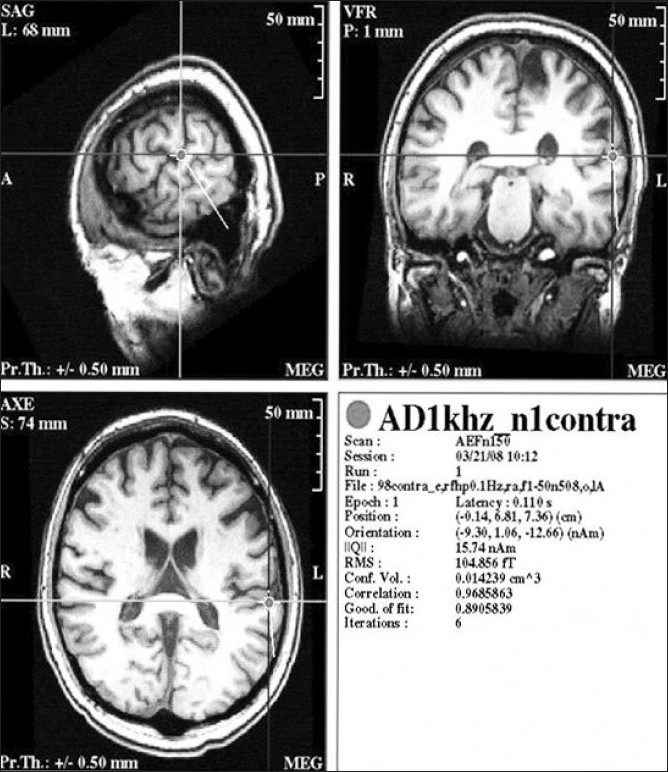
Magnetic evoked response to auditory stimulation localized to the left auditory cortex

Cortical localization of auditory [Figure 4] and visual function using MEG, are well described.[53,54] The MEG detection of brain responses to auditory stimulation have been shown to be consistent and exceedingly sensitive for detection of cortical abnormalities. The most prominent peak on which auditory mapping is based is located at approx 100 ms after the onset of stimuli. This is usually localized on the superior temporal gyrus and is typically larger in amplitude and slightly earlier in cortex contra-lateral to the stimulation.

## Localization of Sensorimotor Cortex

**Figure 5:**
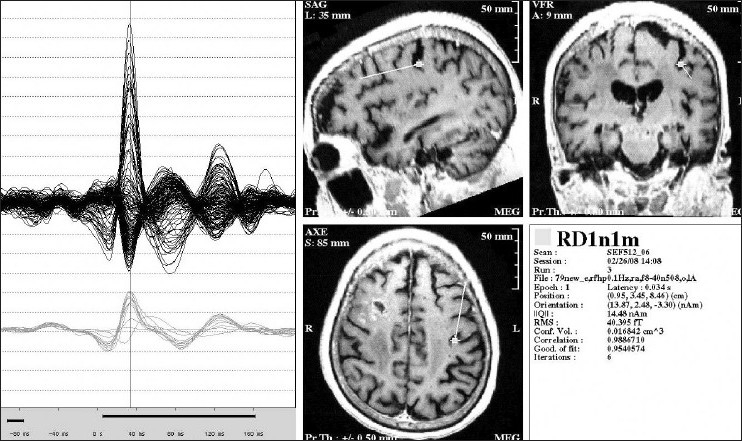
Magnetic evoked response to sensory stimulation of the right thumb with an air driven pressure pulse tapper that taps the thumb surface. Evoked response localizes to the anterior wall of the post-central gyrus. The panel on the left shows the evoked response and the panel on the right shows the ECD localization at the point the cursor has been placed

One of the most common uses of MEG in the functional localization domain is to detect the somatosensory cortex. The somatosensory cortex can be mapped by successive tactile stimulation of fingers, toes, and lips using an electrical stimulator. As most MEG labs have trouble using an electric stimulator so we use a pressure pulse tapper, which is a plastic stimulator that can be clipped on any toe, finger, or lip. The stimulating electrodes are placed on the various peripheral nerves (e.g. median, tibial, etc), and the intensity set such that muscle twitching is barely elicited. Brain magnetic potentials in response to successive stimuli applied to the finger or toe are recorded. With median nerve electrical stimulation, the early N20 component of the evoked magnetic field is easily detected in nearly all patients. The N20 generator is normally located in the anterior wall of the postcentral gyrus [Figure 5] with a tangential orientation, well suited for detection with MEG. Usually, the primary somatosensory cortex is localized by determining an ECD model location of the N20. MEG identification of the somatosensory cortex has been validated by several groups using intraoperative measurements.[55,56] Firsching *et al*.[57] reported that in 30 patients, ECD modeling for the MEG potential in response to tactile stimulation was localized to the somatosensory cortex in all patients and this localization was always in agreement with phase reversal measured at the time of surgery by electrocorticography (ECoG).

The MEG can also be used for localization of the motor cortex.[58,59] The high temporal and spatial resolution of this technique again offers significant advantages in motor mapping as compared to fMRI techniques, as the successive activation of other adjacent areas for instance somatosensory cortex (in addition to the primary motor cortex) can be appreciated. The primary motor cortex is usually identified by localizing the MEG potential that peaks between 20 and 50 ms before the onset of movement, as measured by electromyogram (EMG) surface electrodes.[60] However, as has been discussed, since motor evoked responses are much more complicated than sensory responses and involves significant contribution from other areas, often the localization is also less accurate. Other techniques like coherence analysis,[61] which essentially measures the connectivity of various brain regions, have been suggested to improve localization; the details however are outside the scope of this review.

## Language Localization Using MEG

MEG is extremely well suited for the purposes of language localization since complex cognitive functions like language involve the sequential activation of multiple areas of the brain. Currently, the intracarotid amobarbital test (IAP), also known as the WADA test, is the most common test for language lateralization although increasingly this is being used less often compared to other techniques like functional MRI (fMRI) and MEG. In addition, intracranial electrical stimulation using implanted electrodes is accepted as the gold standard for localization of language cortex. However, both the WADA and the intracranial electrode stimulation are invasive techniques that entail a certain risk of morbidity. The MEG is increasingly being recognized as a tool for non-invasive lateralization as well as localization of language cortex.

In our laboratory, we have typically used MR-FOCUSS, which is a current density imaging technique, for detection of language function.[62–64] This technique allows for the detection of specific cortical areas involved in language processing and the time course of neuronal activation connecting these areas. Current distribution techniques provide an extended cortical view of brain region activation over the more rudimentary ECD analysis that assumes that the magnetic field can be mathematically treated as though it were produced by a simple single point source. The ECD technique is thus likely to have significant limitations when used to analyze complex cortical processes such as language where many regions of the brain are simultaneously active. However, some groups have used this technique for lateralization of language function.[65,66] In contrast, MR-FOCUSS or the minimum norm techniques can provide localizations of multiple simultaneously activated cortical sites, and thus include all cortical activations at each instant.

**Figure 6:**
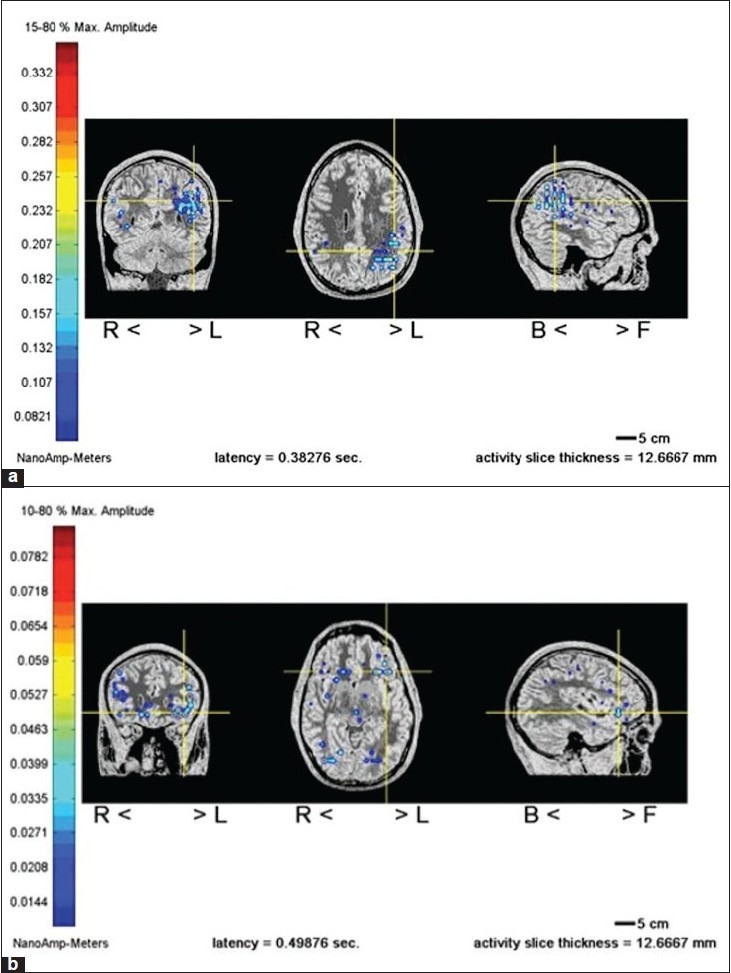
(a and b) MR-FOCUSS localization of language function. Demonstrates localization of receptive language in left hemisphere Wenicke's area for a verb generation task. Demonstrates localization of expressive language in left hemisphere Broca's area for a picture naming task

Using the MR-FOCUSS technique we have found that MEG signals arising from activation of Wernicke's area of the dominant language [Figure 6] hemisphere occurred at a latency of 230-290 milliseconds (ms) after the onset of the language stimulus (typically a verb generation task), while activation was seen in Broca's area at 390 to 460 ms after stimulus onset. [62] In addition, activation of the basal temporal language area was also noted at 150-185 ms.[64]

In another study of 27 patients, performed at our facility, who had WADA testing as well as an MEG study for language lateralization, the MEG (at Broca's area latency) and WADA were in agreement in 23 of 24 (96%) patients who had a successful WADA test performed.[63] In addition, the MEG (at Broca's area latency) correctly lateralized, as was determined by subsequent ECoG, one of three patients who had an undetermined or bilateral IAP. These results indicate an 89% agreement rate (24 of 27) for magnetoencephalographic determination of the hemisphere of language dominance. These data are consistent with those obtained by other investigators using more traditional ECD techniques,[65,66] which mention an overall concordance of MEG with Wada at around 90%.

In conclusion, MEG has inherent advantages, aside from just the non-invasive nature of this test, for detection of language function. This includes a significantly higher temporal resolution than fMRI: milliseconds as opposed to seconds. fMRI records vascular changes occurring over an 8second s interval, resulting in static images that include critical and noncritical language localizations.[62] fMRI also carries the potential risk of providing displaced localizations when abnormalities of vasculature are present, such as arteriovenous malformations (AVM). In contrast, each MEG image measures cerebral neuronal activation with millisecond time resolution over the entire length of the magnetic evoked response, thus allowing for systematic evaluation of sequential steps involved in language function.

Relatively smaller studies of MEG in evaluating memory function have also been performed. Depending on the results of future studies in this area, it is not difficult to conceive that MEG may replace the WADA test for purposes of language and memory lateralization.

## Conclusions

MEG is a useful technique with many recognized and potential applications. MEG could be used to complement the EEG for localization of the seizure focus as it has inherent advantages over the latter. The combination of non-invasiveness with extremely high spatial and temporal resolution is unmatched as compared to other available techniques. The overall accuracy of MEG source localization is better than the EEG. In addition, the fact that the MEG is not contaminated by purely radial sources makes it an easier source to model. Apart from localization of the epileptic focus, functional localization in the brain cortex is likely to be the more important application of this technique in the future. In addition to relatively simple functions like sensation and vision, the MEG can be used to delineate much more complex cognitive processes such as language and memory and thus have the potential to change current paradigms used for localization of eloquent cortex.
